# Liquid Cladding Mediated Optical Fiber Sensors for Copper Ion Detection

**DOI:** 10.3390/mi9090471

**Published:** 2018-09-17

**Authors:** Vien Thi Tran, Nhu Hoa Thi Tran, Than Thi Nguyen, Won Jung Yoon, Heongkyu Ju

**Affiliations:** 1 Department of Nano-Physics, Gachon University, Seongnam-si 461-701, Korea; tranvien04@gmail.com (V.T.T.); ttnhoa@hcmus.edu.vn (N.H.T.T.); nguyenthan1093@gmail.com (T.T.N.); 2 Gachon Bionano Research Institute, Gachon University, Seongnam-si 461-701, Korea; 3 Department of Chemical and Bio Engineering, Gachon University, Seongnam-si 461-701, Korea; wjyoon@gachon.ac.kr; 4 Neuroscience Institute, Gil Hospital, Incheon 405-760, Korea

**Keywords:** heavy metal detection, fiber sensor, liquid cladding, surface chemistry, waveguide numerical aperture

## Abstract

We present a label-free optical fiber based sensor device to detect copper ions (Cu^2+^) in water. A multimode optical fiber, with its polymer cladding removed along a 1-cm length, is used for the optical sensor head, where the injected Cu^2+^ in the liquid phase acts as a liquid cladding for the optical mode. The various Cu^2+^ concentrations modulate the numerical aperture (NA) of the liquid cladding waveguide part. The degree of NA mismatch between the liquid cladding and solid cladding guided parts gives rise to an optical power transmittance change, forming the sensing principle. The presented liquid cladding fiber sensor exhibits a minimum resolvable refractive index of 2.48 × 10^−6^. For Cu^2+^ detection, we functionalize the sensor head surface (fiber core) using chitosan conjugated ethylenediaminetetraacetic acid (EDTA) which captures Cu^2+^ effectively due to the enhanced chelating effects. We obtain a limit of detection of Cu^2+^ of 1.62 nM (104 ppt), which is significantly lower than the tolerable level in drinking water (~30 µM), and achieve a dynamic range of 1 mM. The simple structure of the sensor head and the sensing system ensures the potential capability of being miniaturized. This may allow for in-situ, highly-sensitive, heavy metal sensors in a compact format.

## 1. Introduction

Copper ion (Cu^2+^) is a transition metal that is critically hazardous for the fundamental physiological system of human beings. This ion and its complexes are important components in various metal–coenzyme systems and nutrients [1,2]. As per World Health Organization (WHO) standards, the threshold limit of Cu^2+^ in drinking water is ~30 µM [3]. However, the presence of an elevated concentration of Cu^2+^ leads to gastrointestinal disturbance, liver and kidney damage, and neurodegeneration disease [2,4,5,6,7]. 

Therefore, numerous methods have been considered as tools for the sensitive detection and monitoring of Cu^2+^, in-vivo and in-vitro, such as inductively coupled plasma (ICP) [8,9], near-infrared up-conversion chemodosimeters, which directly detect Cu^2+^ in vivo [10], electrochemical [11], fluorescence [12,13], and colorimetric [14,15]. Magnetic nanoparticle (NMP)-based magnetic resonance imaging (MRI) has also been carried out for Cu^2+^ detection [16]. There is a variety of optical platforms for heavy metal detection, such as surface plasmon resonance (SPR) [17,18,19], optical interferometric sensors [20,21], and surface-enhanced Raman scattering (SERS)-based optical fiber [22]. In particular, the use of optical fiber sensors supports rapid, on-site, data-acquisition, as well as cost-effectiveness, flexibility, size compactness, and remote sensing capacity [21,23].

Recently, label-free optical sensors with liquid-cladding modulation have been demonstrated for highly sensitive detection of analytes in a liquid phase using a multimode optical fiber without additional coatings of layers, such as plasmonic nanometer-thick metals [24]. Analyte liquid injected onto the fiber core acts as a liquid cladding of the fiber, where the original solid cladding was removed. Injection of different concentrations of liquid analyte, which have different refractive indexes, cause the waveguide numerical aperture (NA) to change across the interface between solid and liquid cladding waveguide parts. This leads optical power transmittance to change at the fiber sensor output as a function of the injected analyte concentration, forming the principle of a highly sensitive refractometer. This cladding modulated sensor platform has characteristics that show that sensitivity does not depend on the sensing length, but rather on NA changes across the interface between the two waveguides.

In this work, we demonstrate a liquid-cladding modulated fiber sensor of only 1 cm in length (much shorter than the 5-cm length in Reference [24]) that still shows a similar sensitivity in the refractive index resolution, and put this sensor platform to use for detecting heavy metal ions, such as Cu^2+^, in water by additional immobilization of ligand layers. Prior to ligand layer immobilization, we used glycerol solutions of various concentrations as test mediums to check the refractive index resolution as a refractometer and obtained an index resolution of 2.48 × 10^−6^ refractive index units (RIU). This is comparable to, or even better than, the plasmonic fiber sensor, despite its short sensor length. For Cu^2+^ detection, we functionalized the fiber core with chitosan-conjugated ethylenediaminetetraacetic acid (chitosan-EDTA) composite. We obtained a limit of detection (LOD) of Cu^2+^ of 1.62 nM with a dynamic range of 1 mM. This optical sensor device shows the potential capacity of in-situ sensitive detection of Cu^2+^ in water in a compact format.

## 2. Materials and Methods

### 2.1. Materials and Reagents

Chitosan (low molecular weight), EDTA (99%), (3-aminopropyl)triethoxysilane (APTES, 98%), isopropanol (C_3_H_8_O, 99.7%), acetic acid (C_2_H_4_O_2_, 99.7%), 1-ethyl-3-(3-(dimethylamino)propyl) carbodiimide (EDC), N-hydroxysuccinimide (NHS, 98%), and copper (II) nitrate trihydrate (Cu(NO_3_)_2_.3H_2_O, 99%) were purchased from Sigma-Aldrich Co. (St Louis, MO, USA). Deionized water was produced by Biosesang Co. (Seongnam-Si, Korea). Glycerol (C_3_H_8_O_3_, 99%) was purchased from Duksan Pure Chemicals Co. (Ansan, Korea), and polydimethylsiloxane (PDMS) was purchased from Dow Corning Co. (Hemlock, ML, USA).

### 2.2. Fiber Sensor Surface

**Figure 1:**
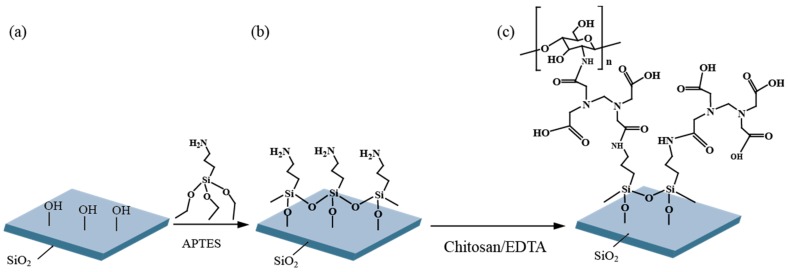
The functionalization of ethylenediaminetetraacetic acid (EDTA) -chitosan on silica surface: (**a**) hydroxyl group (-OH) generated on silica core surface by an oxygen plasma; (**b**) the amine group created via the silanization with (3-aminopropyl)triethoxysilane (APTES); (**c**) the EDTA-chitosan layer self-assembled through amine-carboxyl bonding.

A multimode optical fiber (0.37 NA, JFTLH-Polymicro Technologies, Molex, Lincolnshire, IL, USA) with fiber core/cladding diameters of 200/230 µm was stripped off the polymer cladding along a 1-cm length using a soldering machine (used to remove both the fiber buffer and the polymer cladding), followed by cleansing with a mixture of ethanol/acetone (volume-to-volume ratio of 1:3). Absence of any significant damage to or contamination of the fiber core could then be confirmed using a microscope. The fiber core of silica was modified using functionalized agents for Cu^2+^ detection, as shown in Figure 1. First, hydroxyl groups (-OH) were generated on the fiber core surface via oxygen plasma treatment. This was done by putting a whole fiber sensor in the chamber of an oxygen plasma machine (Cute, Femto Science Inc., Hwaseong-Si, Korea) and running it for 3 min with 20 standard cubic centimeters per minute (sccm) of O_2_ gas. The surface was then immersed in APTES solution (2% APTES in isopropanol) for 24 h at room temperature to produce amine group silanization. The surface was rinsed with isopropanol and dried in a vacuum oven at 60 °C for 4 h. A chitosan solution of 0.08% was prepared by completely dissolving chitosan powder in 0.16% acetic acid solution, using a magnetic stirrer at room temperature for 24 h.

The carboxyl groups of the 0.05 M EDTA solution were activated by 0.1 M EDC and 0.2 M NHS, prior to its conjugation with chitosan. The amine-modified fiber core surface was then immersed in the prepared chitosan-conjugated EDTA at room temperature for 4 h. The chitosan conjugation was expected to enhance the chelating effects of EDTA for capturing Cu^2+^.

**Figure 2:**
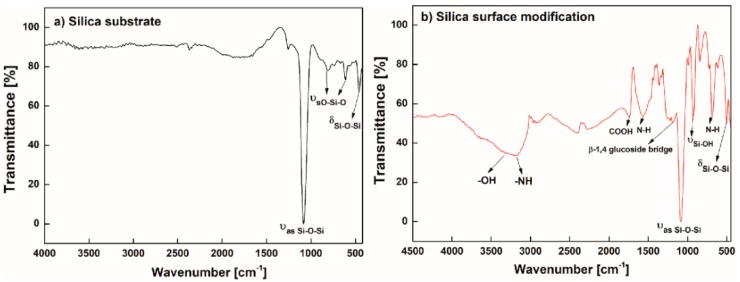
Fourier-transform infrared spectroscopy (FTIR) spectra of: (**a**) Silica substrate; (**b**) chitosan-conjugated EDTA treated silica surface.

The final functionalization of the silica core surface was indirectly checked using an alternative silica substrate surface, onto which the same protocols for functionalization were applied, and was verified using Fourier-transform infrared spectroscopy (FTIR) (PerkinElmer, Waltham, MA, USA). Figure 2a shows the FTIR spectrum for the silica surface of the fiber core. It was revealed that the Si-O-Si asymmetric stretching vibration mode was at 1074 cm^−1^ and the Si-O-Si bending vibration mode was at 456 cm^−1^ [24,25]. In addition, symmetric stretching vibration mode O-Si-O was exhibited at 821 cm^−1^ and 600 cm^−1^ [25,26], indicating the presence of all vibration-mode relevant silica. Figure 2b shows the FTIR spectrum of the silica core surface functionalized with chitosan-conjugated EDTA. It involved silica-related modes, such as the Si-O-Si asymmetric stretching vibration mode at 1084 cm^−1^, the Si-O-Si bending vibration mode at 496 cm^−1^ and the O-Si-O symmetric stretching vibration mode at 838 cm^−1^. We also observed a broadband signal in the range of 3200–3600 cm^−1^ for the vibration mode of an OH group, which overlapped with a stretching vibration mode of N-H [27]. The out-of-plane and in-plane bending vibrations of N-H were exhibited at 680 cm^−1^ and 1566 cm^−1^, respectively [24,28]. We observed the stretching vibration mode of a carboxyl (COOH) group at 1720 cm^−1^ [29], and the stretching vibration mode of silanol (Si-OH mode) at 926 cm^−1^, indicating the silanization of APTES on the silica surface [25]. The presence of chitosan was also confirmed by the presence of a β-1,4 glucoside bridge [27]. All the vibration modes had characteristic bands of either EDTA or chitosan.

**Figure 3:**
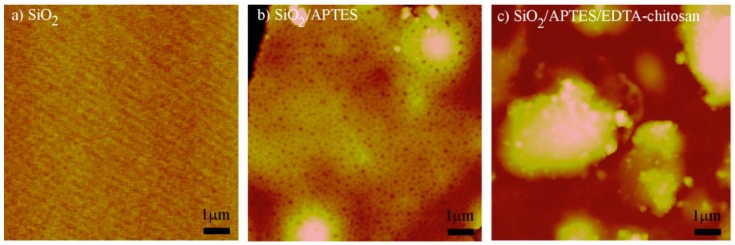
Atomic force microscope (AFM) image of (**a**) silica surface; (**b**) APTES-treated silica surface; (**c**) surface functionalized with chitosan conjugated EDTA.

**Figure 4:**
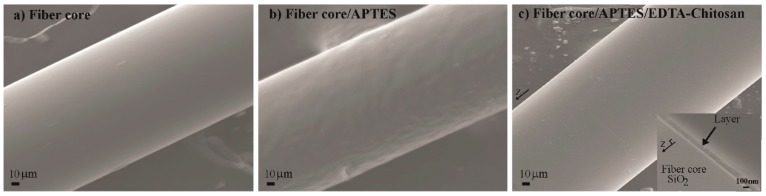
Scanning electron microscopy (SEM) images of (**a**) optical fiber core; (**b**) fiber core treated with APTES; (**c**) fiber core/APTES functionalized with chitosan conjugated EDTA (the inset image is a part of fiber cross-section that contains parts of immobilized layers and the fiber core).

Figure 3a–c shows atomic force microscope (AFM) images for the morphologies of the three surfaces, i.e., the original silica surface, the subsequently APTES-treated surface, and the surface with functionalization of chitosan-conjugated EDTA added on the APTES-treated surface. It was seen that the roughness was 0.168 nm, 15.7 nm, and 63.48 nm for the three surfaces, respectively. The different roughness indicated the different nature of the surface morphology. It was also seen in Figure 3c that there was a heterogeneity of surface morphology. This was due to the possibility of vertical stacking of chitosan-conjugated EDTA layers, as well as the horizontal covering of such layers. However, this homogeneity did not play a detrimental role in sensor performance due to its vertical dimension variation, which is still much smaller than the decay length (about 200 nm) of an evanescent light field. Scanning electron microscopy (SEM) images of the three surfaces are shown in Figure 4a–c. The thickness of a layer of chitosan-conjugated EDTA on an optical fiber was observed to be ~125 nm (the inset image of Figure 4c). The chemical functionalization thickness, which was much smaller than the visible wavelength, set the baseline of a refractive index as the refractometer sensing surface before injecting analyte solutions of various concentrations.

### 2.3. Measurement Setup

**Figure 5:**
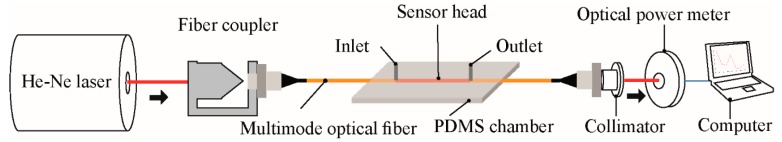
Experimental setup for the fiber-based sensor system.

The sensor head was housed in a PDMS chamber, which allowed to feed and drain liquid (50 µL volume) via two ports, i.e., inlet and outlet under the push of a pump (SMP-21, EYELA, Clifton, NJ, USA). Figure 5 shows the experimental setup for the Cu^2+^ sensor system. A He-Ne laser (632.8 nm), used as a light source, was coupled to one end of the fiber sensor device via in-coupling optics. The light passing through the sensor head was then coupled out of the other end of the fiber and the output optical power was measured using an optical power meter (PM 100D, Thorlabs, Newton, NJ, USA) interfaced with a computer, which recorded the data.

## 3. Results and Discussion

### 3.1. Detection of Glycerol as Test Media

First, as test media, we used solutions of glycerol in water with concentrations ranging from 0 to 30% (volume-to-volume ratio) to test the fiber sensor device’s sensitivity. For prior determination of refractive indices of the glycerol solutions at various concentrations, we used a commercialized refractometer, an Abbe refractometer (DR-A1), which had an index resolution of 10^−4^ refractive index (RI); their measurements are shown in Table 1, with a fitting curve of *y* = 1.33314 + 0.0152. C_g_ (R^2^ = 0.99984). The fitted curve was used to convert the concentration to the corresponding refractive index.

**Figure 6:**
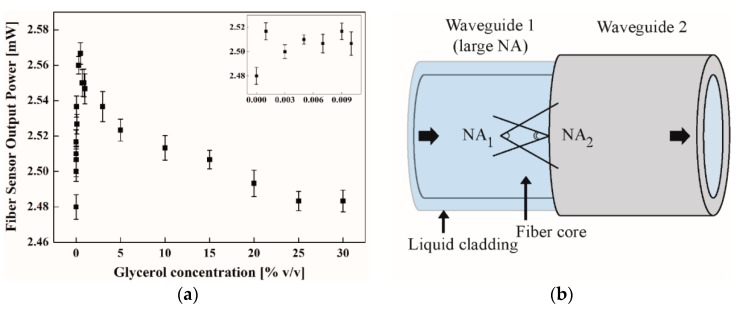
(**a**) Fiber sensor output power with varying concentrations of glycerol (0 to 30% *v*/*v*). (**b**) The interface of the two waveguide domains and the numerical aperture (NA) mismatch between the two.

Prior to surface functionalization with APTES/chitosan-EDTA, we measured the power transmittance response of the device, consisting of core and liquid cladding (glycerol solution, Sigma-Aldrich Co.), as we injected glycerol solutions in various concentrations. Similar to a previous report [24], we obtained a linear relationship between the glycerol concentration and the output power in a low concentration range, as shown in Figure 6a. The mechanism of NA mismatch at the interface between the liquid cladding waveguide and the solid cladding waveguide came into play for optical power increases with increasing concentrations, as depicted in Figure 6b. Waveguide NA = $\sqrt{n_{\mathrm{core}}^{2}-n_{\mathrm{clad}}^{2}}$, where *n*_core_ and *n*_clad_, were the indices of the core and cladding, respectively. When connecting two waveguides of different NAs, optical power transmittance may change. If the first waveguide NA was smaller than the second one, the optical energy transmitted without loss (full transmittance). However, when the first NA was larger than the second one, part of the optical energy was blocked across the interface due to optical mode energy redistribution under more restricted degrees of freedom, given by smaller NAs. With an increase in NA mismatch, the transmitted optical energy decreased, according to T ~(NA_2_/NA_1_)^2^ for NA_1_ > NA_2_ where T denotes the transmittance [30]. Accordingly, under the injection of a zero concentration (deionized water), the liquid cladding waveguide had a much larger NA than that of the polymer (solid) cladding fiber waveguide, leading to a small transmittance of power. Hence, a higher concentration of injected glycerol produces a higher index liquid cladding, and decreases the NA of the liquid cladding waveguide, thus NA mismatch was reduced across the interface between the two kinds of waveguide parts. As shown in Figure 6a, this led to an optical power increase with increasing glycerol concentration near zero. As concentration rose beyond zero, the power decreased modestly due to the evanescent field absorption, which became significant with increasing concentrations, as shown in Figure 6a [24].

It was found that the lowest detectable concentration of glycerol was 3.5 × 10^−3^%, which corresponded to the minimum resolvable refractive index of 2.48 × 10^−6^ RIU. This sensitivity was comparable to, or even better than, those obtained using plasmonic fiber sensors [31,32,33,34,35]. The fact that the sensitivity, which was also of the same order of magnitude as that found in Reference [24], confirmed that the sensor length was not the most important factor for the sensitivity in this kind of sensor device.

### 3.2. Detection of Cu^2+^

Prior to Cu^2+^ detection using the presented fiber sensor device, we checked the indices of the prepared Cu^2+^ solutions, with concentrations from 0 to 2 mM, using a commercialized Abbe refractometer. It was noted that, below a concentration of 100 µM, the refractive indices of Cu^2+^ solutions could not be resolved (not distinguishable from DI water) due to the limited resolution of 10^−4^ RIU.

We employed the presented fiber sensor device with chitosan-conjugated EDTA functionalized on its surface for Cu^2+^ detection at various concentrations. The time-to-data acquisition at a single concentration was about 7 min. We rinsed the sensing surface with DI water to remove non-specific bonding (non-ligand related bonding) before each concentration injection. It was noted that, at each Cu^2+^ concentration injection, the total concentration of Cu^2+^ injected onto the surface needed to be estimated in the aggregate, including those injected precedingly.

**Figure 7:**
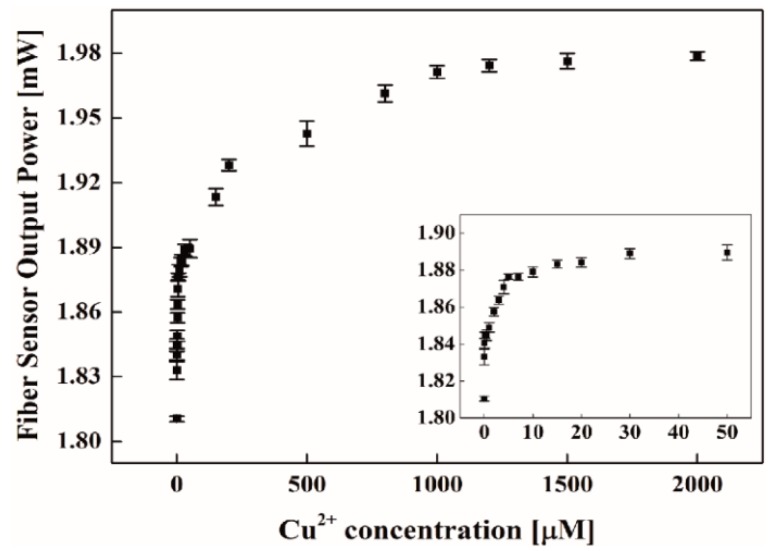
The fiber sensor device output power vs. Cu^2+^ concentrations (0 to 2 mM) with APTES/chitosan-EDTA functionalization on the surface.

As shown in Figure 7, it was revealed that the sensor output power drastically increased with increasing concentrations of Cu^2+^ in its low concentration range. This feature greatly favored ultrasensitive detection of low concentrations of Cu^2+^. Chitosan-conjugated EDTA, which produced strong multi-dentate ligands with enhanced chelating effects, permitted Cu^2+^ to be captured effectively enough for the effective surface index to increase. The consequent complexes of coordinate bonds with Cu^2+^ reduced the NA mismatch at the interface between the two waveguide parts with increasing Cu^2+^ concentrations. At about 10 μM, the output power increase began to saturate, reflecting an effective rise in the liquid cladding index to saturate due to the limited interaction dimensions (a few hundred nanometers in surface normal) between Cu^2+^ and evanescent electromagnetic fields, which decayed in the cladding region from the sensing surface. Unlike the glycerol measurement results, injection of higher concentrations of Cu^2+^ showed no decrease in output power, indicating little absorption of evanescent light by the coordinate bond complexes with Cu^2+^ in the liquid cladding region.

We obtained a dynamic range of 1 mM and a LOD of 1.62 nM (104 ppt) for Cu^2+^ detection in water, which was much lower than the WHO standard (30 µM) for drinking water (LOD was defined as the concentration corresponding to three-times the standard deviation of a blank signal). This LOD was also lower than, or comparable to, recently reported sensors, such as the colorimetric sensors (0.02 µM) [36], the fluorescence sensors (6 nM, 50 nM) [37,38], and colorimetric/fluorescent sensors (0.73 nM) [39]. It should also be mentioned that use of the chitosan-conjugated EDTA did not permit its reusability for this sensor, nor distinguishability of Cu^2+^ from other heavy metal ions. Nevertheless, the presented liquid-cladding fiber sensor showed a great capacity for heavy metal detection, such as Cu^2+^, in the aqueous phase, being able to be used as a powerful tool for in-situ, highly-sensitive detection of heavy metals in an environment, in a compact format.

## 4. Conclusions

We demonstrated a liquid-cladding-modulated, optical-fiber sensor for Cu^2+^ detection. Analyte in the liquid phase injected onto the core surface of an optical fiber that was formerly stripped off its polymer cladding acts as liquid cladding in the fiber sensor head. Different concentrations of analytes change the waveguide NA. This caused a change in optical power transmittance through the interface between the liquid cladding and the solid cladding guided parts, forming the mechanism for a highly sensitive refractometer. We demonstrated a minimum resolvable refractive index of 2.48 × 10^−6^ RIU with this liquid cladding fiber sensor. Chitosan-conjugated EDTA was further functionalized on the fiber core surface to enhance the chelating effects to capture Cu^2+^ more effectively. We achieved a dynamic range of 1 mM and LOD of Cu^2+^ of 1.62 nM (104 ppt), which was encouragingly low, showing the capability of in-situ, highly-sensitive detection of heavy metal ions, such as Cu^2+^, in water, in a compact device. Further optimization may include the use of a separate reference signal to monitor the signal power noise and to subtract the noise properties from the signal noise for enhancing the LOD of the sensor, which relies on optical power measurements.

## Figures and Tables

**Table 1:** The refractive indices measured for various glycerol concentrations (C_g_).

C_g_ (% *v*/*v*)	0	0.005	0.01	0.05	0.1	0.5	1	5	10	20	30
RI (a.u.)	1.3331	1.3331	1.3332	1.3333	1.3334	1.3336	1.3346	1.341	1.3479	1.3634	1.3785

## References

[1] Bost M., Houdart S., Oberli M., Kalonji E., Huneau J.F., Margaritis I. (2016). Dietary copper and human health: Current evidence and unresolved issues. J. Trace Elem. Med. Biol..

[2] Cerpa W., Varela-Nallar L., Reyes A.E., Minniti A.N., Inestrosa N.C. (2005). Is there a role for copper in neurodegenerative diseases?. Mol. Asp. Med..

[3] Fitzgerald D.J. (1998). Safety guidelines for copper in water. Am. J. Clin. Nutr..

[4] Haywood S. (1980). The effect of excess dietary copper on the liver and kidney of the male rat. J. Comp. Pathol..

[5] Waggoner D.J., Bartnikas T.B., Gitlin J.D. (1999). The role of copper in neurodegenerative disease. Neurobiol. Dis..

[6] Giampietro R., Spinelli F., Contino M., Colabufo N.A. (2018). The pivotal role of copper in neurodegeneration: A new strategy for the therapy of neurodegenerative disorders. Mol. Pharm..

[7] Honda R., Nogawa K. (1987). Cadmium, zinc and copper relationships in kidney and liver of humans exposed to environmental cadmium. Arch. Toxicol..

[8] Wu J., Boyle E.A. (1997). Low blank preconcentration technique for the determination of lead, copper, and cadmium in small-volume seawater samples by isotope dilution ICPMS. Anal. Chem..

[9] Hare D.J., Lee J.K., Beavis A.D., Van Gramberg A., George J., Adlard P.A., Finkelstein D.I., Doble P.A. (2012). Three-Dimensional atlas of iron, copper, and zinc in the mouse cerebrum and brainstem. Anal. Chem..

[10] Liu Y., Su Q., Chen M., Dong Y., Shi Y., Feng W., Wu Z.-Y., Li F. (2016). Near-Infrared upconversion chemodosimeter for in vivo detection of Cu^2+^ in wilson disease. Adv. Mater..

[11] Yang Y., Ibrahim A.A., Hashemi P., Stockdill J.L. (2016). Real-Time, selective detection of copper(II) using ionophore-grafted carbon-fiber microelectrodes. Anal. Chem..

[12] Jin L.H., Han C.S. (2014). Ultrasensitive and selective fluorimetric detection of copper ions using thiosulfate-involved quantum dots. Anal. Chem..

[13] Yang P., Zhao Y., Lu Y., Xu Q.-Z., Xu X.-W., Dong L., Yu S.-H. (2011). Phenol formaldehyde resin nanoparticles loaded with CdTe quantum dots: A fluorescence resonance energy transfer probe for optical visual detection of copper(II) ions. ACS Nano.

[14] Ma Y.R., Niu H.Y., Cai Y.Q. (2011). Colorimetric detection of copper ions in tap water during the synthesis of silver/dopamine nanoparticles. Chem. Commun..

[15] Gao Q., Ji L., Wang Q., Yin K., Li J., Chen L. (2017). Colorimetric sensor for highly sensitive and selective detection of copper ion. Anal. Methods.

[16] Yin H., Kuang H., Liu L., Xu L., Ma W., Wang L., Xu C. (2014). A ligation dnazyme-induced magnetic nanoparticles assembly for ultrasensitive detection of copper ions. ACS Appl. Mater. Interface.

[17] Fen Y.W., Yunus W.M.M., Yusof N.A. (2011). Detection of mercury and copper ions using surface plasmon resonance optical sensor. Sens. Mater..

[18] Palumbo M., Pearson C., Nagel J., Petty M.C. (2003). Surface plasmon resonance sensing of liquids using polyelectrolyte thin films. Sens. Actuators B Chem..

[19] Chen H., Jia S., Zhang J., Jang M., Chen X., Koh K., Wang Z. (2015). Sensitive detection of copper(II) ions based on the conformational change of peptides by surface plasmon resonance spectroscopy. Anal. Methods.

[20] Yao B.C., Wu Y., Yu C.B., He J.R., Rao Y.J., Gong Y., Fu F., Chen Y.F., Li Y.R. (2016). Partially reduced graphene oxide based FRET on fiber-optic interferometer for biochemical detection. Sci. Rep..

[21] Raghunandhan R., Chen L.H., Long H.Y., Leam L.L., So P.L., Ning X., Chan C.C. (2016). Chitosan/PAA based fiber-optic interferometric sensor for heavy metal ions detection. Sens. Actuators B Chem..

[22] Cheng F., Xu H., Wang C., Gong Z., Tang C., Fan M. (2014). Surface enhanced Raman scattering fiber optic sensor as an ion selective optrode: The example of Cd^2+^ detection. RSC Adv..

[23] Ji W.B., Yap S.H.K., Panwar N., Zhang L.L., Lin B., Yong K.T., Tjin S.C., Ng W.J., Majid M.B.A. (2016). Detection of low-concentration heavy metal ions using optical microfiber sensor. Sens. Actuators B Chem..

[24] Tran N.H.T., Kim J., Phan T.B., Khym S., Ju H. (2017). Label-Free optical biochemical sensors via liquid-cladding-induced modulation of waveguide modes. ACS Appl. Mater. Interface.

[25] Dattelbaum A.M., Amweg M.L., Ruiz J.D., Ecke L.E., Shreve A.P., Parikh A.N. (2005). Surfactant removal and silica condensation during the photochemical calcination of thin film silica mesophases. J. Phys. Chem. B.

[26] Al-Oweini R., El-Rassy H. (2009). Synthesis and characterization by FTIR spectroscopy of silica aerogels prepared using several Si(OR)_4_ and R”Si(OR’)_3_ precursors. J. Mol. Struct..

[27] Fujita S., Sakairi N. (2016). Water soluble EDTA-linked chitosan as a zwitterionicflocculant for pH sensitive removal of Cu(II) ion. RSC Adv..

[28] Ren Y., Abbood H.A., He F., Peng H., Huang K. (2013). Magnetic EDTA-modified chitosan/SiO_2_/Fe_3_O_4_ adsorbent: Preparation, characterization, and application in heavy metal adsorption. Chem. Eng. J..

[29] Pawlak A., Mucha M. (2003). Thermogravimetric and FTIR studies of chitosan blends. Thermochim. Acta.

[30] Senior J.M., Jamro M.Y. (2009). Optical Fiber Communications Principles and Practice.

[31] Nu T.T.V., Tran N.H.T., Nam E., Nguyen T.T., Yoon W.J., Cho S., Kim J., Chang K.-A., Ju H. (2018). Blood-based immunoassay of tau proteins for early diagnosis of Alzheimer’s disease using surface plasmon resonance fiber sensors. RSC Adv..

[32] Nguyen T.T., Trinh K.T.L., Yoon W.J., Lee N.Y., Ju H. (2017). Integration of a microfluidic polymerase chain reaction device and surface plasmon resonance fiber sensor into an inline all-in-one platform for pathogenic bacteria detection. Sens. Actuators B Chem..

[33] Kim J., Kim S., Nguyen T.T., Lee R., Li T., Yun C., Ham Y., An S.S.A., Ju H. (2016). Label-free quantitative immunoassay of fibrinogen in alzheimer disease patient plasma using fiber optical surface plasmon resonance. J. Electron. Mater..

[34] Nguyen T.T., Bea S.O., Kim D.M., Yoon W.J., Park J.W., An S.S.A., Ju H. (2015). A regenerative label-free fiber optic sensor using surface plasmon resonance for clinical diagnosis of fibrinogen. Int. J. Nanomed..

[35] Nguyen T.T., Lee E.C., Ju H. (2014). Bimetal coated optical fiber sensors based on surface plasmon resonance induced change in birefringence and intensity. Opt. Express.

[36] Kim M.S., Lee S.Y., Jung J.M., Kim C. (2017). A new Schiff-base chemosensor for selective detection of Cu^2+^ and Co^2+^ and its copper complex for colorimetric sensing of S^2−^ in aqueous solution. Photochem. Photobiol. Sci..

[37] Dong Y., Wang R., Li G., Chen C., Chi Y., Chen G. (2012). Polyamine-functionalized carbon quantum dots as fluorescent probes for selective and sensitive detection of copper ions. Anal. Chem..

[38] Yin K., Wu Y., Wang S., Chen L. (2016). A sensitive fluorescent biosensor for the detection of copper ion inspired by biological recognition element pyoverdine. Sens. Actuators B Chem..

[39] Chandra R., Ghorai A., Patra G.K. (2018). A simple benzildihydrazone derived colorimetric and fluorescent ‘*on–off-on’* sensor for sequential detection of copper(II) and cyanide ions in aqueous solution. Sens. Actuators B Chem..

